# Supplementary material for: Selected wetland soil properties correlate to Rift Valley fever livestock mortalities reported in 2009-10 in central South Africa
Source: PLoS One. 2020 May 18;15(5):e0232481. doi: 10.1371/journal.pone.0232481 (PMC7233588; doi:10.1371/journal.pone.0232481)
Supplement: S1 Table — (DOCX) [file pone.0232481.s001.docx]

S1 Table Descriptive statistics of the physical and chemical properties by soil layer(where the “Reported” group consists of sites where RVF mortalities have been reported and “Not Reported” consists of sites where RVF mortalities have not been reported).

| Variable | |  | Layers | | | | | | | |
| --- | --- | --- | --- | --- | --- | --- | --- | --- | --- | --- |
|  |  |  | 0-50 mm | | A horizon | | B/B1/G/C1 horizon | | B2/C/C2 horizon | |
|  |  |  | Reported | Not reported | Reported | Not reported | Reported | Not reported | Reported | Not reported |
|  | | Count: | 12 | 10 | 12 | 10 | 12 | 10 | 11 | 7 |
| Soluble cations (cmol_c_ kg^-1^) | Ca^2+^ | Mean | 0.16 | 0.57 | 0.19 | 0.54 | 0.16 | 0.37 | 0.10 | 0.32 |
|  |  | Std. Dev. | 0.12 | 0.37 | 0.28 | 0.43 | 0.22 | 0.41 | 0.08 | 0.19 |
|  |  | Min | 0.01 | 0.20 | 0.03 | 0.20 | 0.03 | 0.12 | 0.02 | 0.10 |
|  |  | Median | 0.14 | 0.54 | 0.12 | 0.41 | 0.08 | 0.24 | 0.08 | 0.31 |
|  |  | Max | 0.35 | 1.49 | 1.06 | 1.57 | 0.83 | 1.49 | 0.27 | 0.60 |
|  | Mg^2+^ | Mean | 0.35 | 0.03 | 0.11 | 0.05 | 0.04 | 0.35 | 0.02 | 0.58 |
|  |  | Std. Dev. | 0.91 | 0.02 | 0.26 | 0.05 | 0.06 | 1.06 | 0.02 | 1.49 |
|  |  | Min | 0.00 | 0.01 | 0.00 | 0.02 | 0.00 | 0.01 | 0.00 | 0.01 |
|  |  | Median | 0.02 | 0.03 | 0.03 | 0.03 | 0.02 | 0.02 | 0.01 | 0.02 |
|  |  | Max | 3.13 | 0.07 | 0.94 | 0.19 | 0.22 | 3.38 | 0.06 | 3.96 |
|  | K^+^ | Mean | 0.02 | 0.01 | 0.00 | 0.01 | 0.00 | 0.02 | 0.00 | 0.02 |
|  |  | Std. Dev. | 0.03 | 0.00 | 0.00 | 0.01 | 0.00 | 0.04 | 0.01 | 0.05 |
|  |  | Min | 0.00 | 0.00 | 0.00 | 0.00 | 0.00 | 0.00 | 0.00 | 0.00 |
|  |  | Median | 0.01 | 0.01 | 0.00 | 0.01 | 0.00 | 0.00 | 0.00 | 0.00 |
|  |  | Max | 0.10 | 0.01 | 0.02 | 0.03 | 0.01 | 0.12 | 0.03 | 0.14 |
|  | Na^+^ | Mean | 7.37 | 0.86 | 1.10 | 1.64 | 0.38 | 1.81 | 0.20 | 1.90 |
|  |  | Std. Dev. | 10.4 | 1.66 | 1.81 | 1.88 | 0.88 | 2.26 | 0.33 | 2.35 |
|  |  | Min | 0.00 | 0.01 | 0.00 | 0.01 | 0.00 | 0.01 | 0.00 | 0.01 |
|  |  | Median | 0.19 | 0.05 | 0.15 | 0.97 | 0.07 | 0.15 | 0.06 | 0.02 |
|  |  | Max | 28.6 | 4.05 | 4.65 | 4.53 | 3.14 | 4.58 | 1.12 | 4.43 |
| Exchangeable cations (cmol_c_ kg^-1^) | Ca^2+^ | Mean | 23.0 | 46.7 | 26.0 | 43.5 | 27.4 | 46.3 | 29.4 | 43.5 |
|  |  | Std. Dev. | 12.3 | 15.5 | 11.5 | 12.5 | 11.4 | 12.0 | 11.3 | 20.0 |
|  |  | Min | 6.50 | 14.2 | 7.15 | 17.5 | 10.3 | 29.4 | 6.96 | 3.53 |
|  |  | Median | 26.1 | 46.8 | 27.7 | 45.1 | 29.4 | 48.0 | 29.7 | 49.9 |
|  |  | Max | 37.8 | 70.5 | 40.4 | 61.0 | 49.8 | 61.5 | 47.5 | 61.4 |
|  | Mg^2+^ | Mean | 7.28 | 12.0 | 6.84 | 10.3 | 7.67 | 11.3 | 9.56 | 12.7 |
|  |  | Std. Dev. | 6.61 | 4.99 | 4.27 | 3.91 | 5.08 | 4.72 | 6.97 | 6.73 |
|  |  | Min | 1.18 | 6.88 | 2.50 | 5.00 | 2.83 | 5.42 | 2.83 | 5.21 |
|  |  | Median | 4.17 | 11.9 | 4.75 | 9.79 | 5.25 | 11.9 | 6.33 | 11.3 |
|  |  | Max | 21.0 | 23.8 | 13.3 | 15.2 | 19.7 | 21.7 | 24.0 | 24.0 |
|  | K^+^ | Mean | 1.94 | 3.12 | 1.54 | 3.01 | 1.60 | 2.89 | 1.36 | 2.79 |
|  |  | Std. Dev. | 0.85 | 1.95 | 0.81 | 1.40 | 0.85 | 1.49 | 0.65 | 2.23 |
|  |  | Min | 1.07 | 0.13 | 0.46 | 0.86 | 0.23 | 0.60 | 0.40 | 0.44 |
|  |  | Median | 1.58 | 2.78 | 1.45 | 2.91 | 1.55 | 2.91 | 1.39 | 2.05 |
|  |  | Max | 3.84 | 6.20 | 2.99 | 5.95 | 3.70 | 5.69 | 2.75 | 7.29 |
|  | Na^+^ | Mean | 2.60 | 4.85 | 2.09 | 9.54 | 1.58 | 8.58 | 1.77 | 9.98 |
|  |  | Std. Dev. | 2.98 | 8.82 | 1.94 | 15.4 | 1.69 | 12.6 | 1.48 | 17.5 |
|  |  | Min | 0.18 | 0.12 | 0.13 | 0.09 | 0.14 | 0.08 | 0.15 | 0.07 |
|  |  | Median | 1.14 | 0.43 | 2.07 | 1.42 | 0.80 | 1.68 | 1.37 | 0.32 |
|  |  | Max | 8.26 | 26.09 | 5.74 | 43.04 | 5.22 | 36.3 | 4.35 | 46.2 |
| CEC | (cmol_c_ kg^-1^) | Mean | 18.9 | 30.3 | 16.4 | 26.3 | 14.8 | 27.6 | 19.6 | 21.7 |
|  |  | Std. Dev. | 11.9 | 8.25 | 9.73 | 9.21 | 5.96 | 11.2 | 9.76 | 18.1 |
|  |  | Min | 3.83 | 18.0 | 4.17 | 16.2 | 4.00 | 15.3 | 5.39 | 1.04 |
|  |  | Median | 15.9 | 32.2 | 13.4 | 25.7 | 15.3 | 26.5 | 20.0 | 14.7 |
|  |  | Max | 40.0 | 41.7 | 36.5 | 40.0 | 22.6 | 47.8 | 36.5 | 52.2 |
| Organic carbon | (mg kg^-1^) | Mean | 36208 | 32560 | 25825 | 21190 | 24442 | 27030 | 27955 | 32029 |
|  |  | Std. Dev. | 24202 | 16614 | 19567 | 8924 | 18461 | 22176 | 20226 | 19522 |
|  |  | Min | 5100 | 10000 | 4100 | 7700 | 5400 | 4800 | 7700 | 6500 |
|  |  | Median | 31350 | 28850 | 19000 | 19850 | 18250 | 20500 | 21100 | 30900 |
|  |  | Max | 76700 | 63900 | 55500 | 42200 | 72200 | 67100 | 67800 | 69100 |
| Total Nitrogen | (mg kg^-1^) | Mean | 2280 | 2448 | 1138 | 1298 | 976 | 687 | 784 | 544 |
|  |  | Std. Dev. | 1447 | 1132 | 667 | 390 | 710 | 262 | 547 | 210 |
|  |  | Min | 300 | 1140 | 200 | 770 | 220 | 330 | 180 | 240 |
|  |  | Median | 2115 | 2045 | 960 | 1200 | 670 | 690 | 570 | 590 |
|  |  | Max | 4740 | 4050 | 2360 | 1790 | 2600 | 1090 | 1920 | 870 |
| Electrical resistance | (Ω) | Mean | 2280 | 2448 | 1138 | 1298 | 976 | 687 | 784 | 544 |
|  |  | Std. Dev. | 1447 | 1132 | 667 | 390 | 710 | 262 | 547 | 210 |
|  |  | Min | 300 | 1140 | 200 | 770 | 220 | 330 | 180 | 240 |
|  |  | Median | 2115 | 2045 | 960 | 1200 | 670 | 690 | 570 | 590 |
|  |  | Max | 4740 | 4050 | 2360 | 1790 | 2600 | 1090 | 1920 | 870 |
| pH_KCl_ |  | Mean | 7.38 | 6.91 | 7.19 | 7.15 | 7.24 | 7.51 | 7.24 | 7.78 |
|  |  | Std. Dev. | 1.95 | 0.86 | 1.47 | 0.93 | 1.09 | 0.92 | 0.88 | 0.80 |
|  |  | Min | 4.15 | 4.86 | 4.65 | 5.05 | 5.16 | 6.01 | 5.50 | 6.30 |
|  |  | Median | 7.50 | 7.16 | 7.42 | 7.37 | 7.49 | 7.61 | 7.50 | 7.80 |
|  |  | Max | 10.0 | 7.75 | 9.35 | 8.21 | 8.82 | 8.84 | 8.54 | 8.91 |
| pH_Water_ |  | Mean | 8.20 | 7.80 | 8.35 | 8.13 | 8.25 | 8.48 | 8.29 | 8.63 |
|  |  | Std. Dev. | 1.57 | 0.89 | 1.16 | 0.94 | 0.83 | 0.86 | 0.75 | 0.77 |
|  |  | Min | 5.44 | 5.75 | 6.37 | 6.28 | 7.11 | 7.38 | 6.96 | 7.80 |
|  |  | Median | 8.01 | 7.88 | 8.16 | 8.07 | 8.18 | 8.19 | 8.31 | 8.34 |
|  |  | Max | 10.4 | 9.08 | 10.0 | 9.46 | 9.77 | 10.0 | 9.64 | 9.98 |
| Coarse sand | (%) | Mean | 3.86 | 5.35 | 3.28 | 4.12 | 3.67 | 5.48 | 2.60 | 6.05 |
|  |  | Std. Dev. | 3.95 | 4.24 | 3.17 | 4.50 | 3.39 | 5.21 | 2.82 | 4.79 |
|  |  | Min | 0.20 | 0.13 | 0.46 | 0.07 | 0.27 | 0.73 | 0.53 | 1.06 |
|  |  | Median | 2.34 | 5.05 | 1.79 | 2.69 | 3.32 | 4.55 | 1.73 | 6.71 |
|  |  | Max | 11.8 | 13.2 | 8.80 | 15.4 | 11.8 | 18.0 | 10.2 | 12.8 |
| Medium sand | (%) | Mean | 4.11 | 6.87 | 5.20 | 7.50 | 4.83 | 7.25 | 3.23 | 6.00 |
|  |  | Std. Dev. | 2.44 | 3.75 | 3.72 | 4.08 | 3.76 | 3.62 | 1.82 | 3.84 |
|  |  | Min | 0.99 | 1.39 | 1.06 | 3.79 | 1.00 | 2.80 | 1.06 | 2.66 |
|  |  | Median | 4.20 | 6.14 | 4.49 | 6.31 | 3.63 | 6.91 | 2.61 | 3.85 |
|  |  | Max | 9.83 | 15.79 | 12.05 | 17.93 | 12.61 | 14.47 | 6.71 | 12.84 |
| Fine sand | (%) | Mean | 22.3 | 25.2 | 24.3 | 27.6 | 19.0 | 26.1 | 17.7 | 21.5 |
|  |  | Std. Dev. | 18.1 | 8.12 | 16.3 | 4.60 | 13.5 | 10.2 | 9.07 | 9.45 |
|  |  | Min | 5.26 | 9.49 | 6.04 | 21.85 | 4.15 | 8.93 | 3.74 | 9.17 |
|  |  | Median | 13.7 | 27.4 | 21.7 | 26.7 | 18.2 | 23.0 | 19.7 | 20.1 |
|  |  | Max | 64.1 | 36.0 | 57.7 | 37.3 | 46.7 | 40.9 | 32.5 | 35.3 |
| Very fine sand | (%) | Mean | 13.0 | 12.9 | 15.3 | 14.8 | 10.9 | 12.3 | 11.1 | 13.3 |
|  |  | Std. Dev. | 6.14 | 3.02 | 7.53 | 2.73 | 5.18 | 3.76 | 4.24 | 5.31 |
|  |  | Min | 7.69 | 7.36 | 7.62 | 11.3 | 4.47 | 6.79 | 4.92 | 6.78 |
|  |  | Median | 11.3 | 13.5 | 13.4 | 14.9 | 8.25 | 13.2 | 11.7 | 14.7 |
|  |  | Max | 28.0 | 16.7 | 35.0 | 20.0 | 20.5 | 18.8 | 19.5 | 21.8 |
| Coarse silt | (%) | Mean | 7.67 | 7.37 | 5.10 | 6.31 | 7.19 | 6.50 | 7.31 | 6.02 |
|  |  | Std. Dev. | 5.05 | 2.19 | 2.03 | 1.98 | 4.24 | 1.60 | 5.12 | 2.85 |
|  |  | Min | 1.84 | 3.46 | 0.99 | 2.70 | 0.90 | 4.63 | 1.89 | 2.06 |
|  |  | Median | 6.66 | 8.17 | 5.03 | 7.10 | 6.34 | 5.70 | 5.62 | 5.58 |
|  |  | Max | 19.90 | 10.10 | 8.97 | 8.33 | 16.10 | 8.94 | 17.80 | 9.34 |
| Fine silt | (%) | Mean | 15.7 | 15.5 | 13.2 | 12.8 | 14.8 | 12.0 | 14.1 | 14.1 |
|  |  | Std. Dev. | 9.52 | 4.70 | 8.64 | 3.59 | 8.89 | 4.66 | 5.25 | 5.07 |
|  |  | Min | 2.59 | 8.19 | 2.55 | 8.20 | 5.16 | 5.61 | 7.70 | 6.78 |
|  |  | Median | 15.1 | 15.9 | 11.2 | 12.9 | 11.5 | 10.6 | 14.0 | 13.1 |
|  |  | Max | 32.7 | 20.8 | 28.9 | 19.7 | 31.8 | 20.1 | 24.8 | 22.1 |
| Clay | (%) | Mean | 32.5 | 23.1 | 29.9 | 22.5 | 37.1 | 27.0 | 42.2 | 28.8 |
|  |  | Std. Dev. | 17.5 | 12.5 | 17.2 | 9.10 | 16.4 | 11.8 | 14.0 | 14.0 |
|  |  | Min | 8.80 | 9.83 | 7.48 | 8.30 | 12.82 | 11.2 | 21.1 | 12.0 |
|  |  | Median | 36.8 | 19.1 | 36.2 | 21.1 | 39.6 | 25.6 | 44.7 | 27.2 |
|  |  | Max | 57.1 | 44.0 | 51.8 | 35.3 | 69.5 | 47.6 | 66.2 | 47.6 |
| Residual silt + clay | (%) | Mean | 2.00 | 2.15 | 1.07 | 1.63 | 2.08 | 2.54 | 1.57 | 1.63 |
|  |  | Std. Dev. | 3.39 | 1.44 | 1.07 | 1.09 | 3.30 | 2.21 | 3.14 | 1.18 |
|  |  | Min | 0.33 | 0.67 | 0.33 | 0.40 | 0.20 | 0.40 | 0.07 | 0.40 |
|  |  | Median | 1.03 | 1.73 | 0.83 | 1.16 | 0.73 | 1.60 | 0.59 | 1.26 |
|  |  | Max | 12.6 | 4.57 | 4.35 | 3.19 | 10.5 | 6.31 | 11.0 | 4.12 |
