# Supplementary material for: Selected wetland soil properties correlate to Rift Valley fever livestock mortalities reported in 2009-10 in central South Africa
Source: PLoS One. 2020 May 18;15(5):e0232481. doi: 10.1371/journal.pone.0232481 (PMC7233588; doi:10.1371/journal.pone.0232481)
Supplement: S2 Table — (DOCX) [file pone.0232481.s002.docx]

S2 Table Descriptive statistics of the soil microbiology and mineralogical analyses (where the “Reported” group are sites where RVF mortalities have been reported and the “Not Reported” group are sites where RVF mortalities have not been reported).

| Variable | Unit |  | Group | |
| --- | --- | --- | --- | --- |
|  |  |  | Reported | Not reported |
| Microbial analysis | | | | |
| Active_C | (mg/kg) | N | 12 | 10 |
|  |  | Mean | 482.33 | 411.16 |
|  |  | Std | 160.34 | 141.06 |
|  |  | Min | 128.34 | 168.93 |
|  |  | Median | 502.70 | 459.08 |
|  |  | Max | 685.04 | 562.94 |
| FDA | (µg FDA/g soil) | N | 12.00 | 10.00 |
|  |  | Mean | 89.14 | 57.09 |
|  |  | Std | 89.38 | 36.04 |
|  |  | Min | 14.23 | 4.76 |
|  |  | Median | 64.54 | 51.63 |
|  |  | Max | 342.28 | 128.59 |
| Clay mineralogical (XRD) analysis | | | | |
| Anatase | (%, m/m) | N | 12.00 | 10.00 |
|  |  | Mean | 0.47 | 0.39 |
|  |  | Std | 1.61 | 1.23 |
|  |  | Min | 0.00 | 0.00 |
|  |  | Median | 0.00 | 0.00 |
|  |  | Max | 5.58 | 3.90 |
| Andalusite |  | N | 12.00 | 10.00 |
|  |  | Mean | 0.74 | 0.00 |
|  |  | Std | 2.55 | 0.00 |
|  |  | Min | 0.00 | 0.00 |
|  |  | Median | 0.00 | 0.00 |
|  |  | Max | 8.85 | 0.00 |
| Ankerite |  | N | 12.00 | 10.00 |
|  |  | Mean | 1.84 | 4.00 |
|  |  | Std | 4.31 | 6.62 |
|  |  | Min | 0.00 | 0.00 |
|  |  | Median | 0.00 | 0.00 |
|  |  | Max | 11.90 | 16.25 |
| Apophyllite |  | N | 12.00 | 10.00 |
|  |  | Mean | 1.04 | 0.00 |
|  |  | Std | 3.61 | 0.00 |
|  |  | Min | 0.00 | 0.00 |
|  |  | Median | 0.00 | 0.00 |
|  |  | Max | 12.51 | 0.00 |
| Calcite |  | N | 12.00 | 10.00 |
|  |  | Mean | 4.52 | 4.82 |
|  |  | Std | 5.07 | 5.67 |
|  |  | Min | 0.00 | 0.00 |
|  |  | Median | 5.19 | 2.91 |
|  |  | Max | 16.58 | 14.22 |
| Dolomite | (%, m/m) | N | 12.00 | 10.00 |
|  |  | Mean | 2.84 | 2.19 |
|  |  | Std | 4.22 | 4.69 |
|  |  | Min | 0.00 | 0.00 |
|  |  | Median | 0.00 | 0.00 |
|  |  | Max | 9.85 | 12.64 |
| Gypsum |  | N | 12.00 | 10.00 |
|  |  | Mean | 1.33 | 0.00 |
|  |  | Std | 4.62 | 0.00 |
|  |  | Min | 0.00 | 0.00 |
|  |  | Median | 0.00 | 0.00 |
|  |  | Max | 16.00 | 0.00 |
| Halite |  | N | 12.00 | 10.00 |
|  |  | Mean | 4.16 | 3.98 |
|  |  | Std | 5.33 | 5.19 |
|  |  | Min | 0.00 | 0.00 |
|  |  | Median | 0.00 | 0.00 |
|  |  | Max | 12.78 | 11.44 |
| K_feldspar_rutile |  | N | 12.00 | 10.00 |
|  |  | Mean | 8.60 | 8.83 |
|  |  | Std | 9.21 | 7.75 |
|  |  | Min | 0.00 | 0.00 |
|  |  | Median | 6.57 | 12.44 |
|  |  | Max | 20.73 | 17.29 |
| Kaolinite |  | N | 12.00 | 10.00 |
|  |  | Mean | 2.69 | 4.92 |
|  |  | Std | 6.28 | 7.96 |
|  |  | Min | 0.00 | 0.00 |
|  |  | Median | 0.00 | 0.00 |
|  |  | Max | 16.13 | 18.16 |
| Mica |  | N | 12.00 | 10.00 |
|  |  | Mean | 24.52 | 24.52 |
|  |  | Std | 9.75 | 9.40 |
|  |  | Min | 0.00 | 0.00 |
|  |  | Median | 24.12 | 25.32 |
|  |  | Max | 35.39 | 34.17 |
| Plagioclase |  | N | 12.00 | 10.00 |
|  |  | Mean | 18.09 | 14.65 |
|  |  | Std | 3.81 | 5.47 |
|  |  | Min | 12.42 | 0.00 |
|  |  | Median | 18.04 | 15.96 |
|  |  | Max | 24.79 | 19.75 |
| Pyroxene |  | N | 12.00 | 10.00 |
|  |  | Mean | 0.00 | 3.19 |
|  |  | Std | 0.00 | 6.72 |
|  |  | Min | 0.00 | 0.00 |
|  |  | Median | 0.00 | 0.00 |
|  |  | Max | 0.00 | 16.22 |
| Quartz | (%, m/m) | N | 12.00 | 10.00 |
|  |  | Mean | 27.04 | 25.66 |
|  |  | Std | 7.86 | 5.80 |
|  |  | Min | 15.26 | 15.79 |
|  |  | Median | 25.69 | 24.34 |
|  |  | Max | 41.15 | 36.29 |
| Smectite |  | N | 12.00 | 10.00 |
|  |  | Mean | 2.12 | 2.84 |
|  |  | Std | 7.35 | 8.99 |
|  |  | Min | 0.00 | 0.00 |
|  |  | Median | 0.00 | 0.00 |
|  |  | Max | 25.47 | 28.42 |
| Total elemental (XRF) analysis | | | | |
| Al_2_O_3_ | (%, m/m) | N | 12.00 | 10.00 |
|  |  | Mean | 7.93 | 8.89 |
|  |  | Std | 3.99 | 3.53 |
|  |  | Min | 2.80 | 5.20 |
|  |  | Median | 7.70 | 7.55 |
|  |  | Max | 13.00 | 13.60 |
| CaO |  | N | 12.00 | 10.00 |
|  |  | Mean | 4.81 | 3.53 |
|  |  | Std | 5.24 | 3.25 |
|  |  | Min | 0.40 | 0.40 |
|  |  | Median | 3.60 | 2.50 |
|  |  | Max | 17.60 | 10.20 |
| Fe_2_O_3_ |  | N | 12.00 | 10.00 |
|  |  | Mean | 4.23 | 4.96 |
|  |  | Std | 1.64 | 1.57 |
|  |  | Min | 1.60 | 3.30 |
|  |  | Median | 4.75 | 4.65 |
|  |  | Max | 6.30 | 8.00 |
| K_2_O |  | N | 12.00 | 10.00 |
|  |  | Mean | 1.42 | 1.58 |
|  |  | Std | 0.62 | 0.36 |
|  |  | Min | 0.70 | 1.20 |
|  |  | Median | 1.30 | 1.45 |
|  |  | Max | 2.40 | 2.20 |
| MgO |  | N | 12.00 | 10.00 |
|  |  | Mean | 2.30 | 2.57 |
|  |  | Std | 1.62 | 1.65 |
|  |  | Min | 0.80 | 0.70 |
|  |  | Median | 2.00 | 2.30 |
|  |  | Max | 6.30 | 6.10 |
| MnO |  | N | 12.00 | 10.00 |
|  |  | Mean | 0.05 | 0.08 |
|  |  | Std | 0.05 | 0.06 |
|  |  | Min | 0.00 | 0.00 |
|  |  | Median | 0.05 | 0.10 |
|  |  | Max | 0.10 | 0.20 |
| Na_2_O | (%, m/m) | N | 12.00 | 10.00 |
|  |  | Mean | 1.00 | 0.61 |
|  |  | Std | 0.67 | 0.34 |
|  |  | Min | 0.30 | 0.30 |
|  |  | Median | 0.85 | 0.50 |
|  |  | Max | 2.10 | 1.40 |
| P_2_O_5_ |  | N | 12.00 | 10.00 |
|  |  | Mean | 0.09 | 0.11 |
|  |  | Std | 0.07 | 0.03 |
|  |  | Min | 0.00 | 0.10 |
|  |  | Median | 0.10 | 0.10 |
|  |  | Max | 0.20 | 0.20 |
| SiO_2_ |  | N | 12.00 | 10.00 |
|  |  | Mean | 64.42 | 66.14 |
|  |  | Std | 12.21 | 7.22 |
|  |  | Min | 49.70 | 58.70 |
|  |  | Median | 62.60 | 65.65 |
|  |  | Max | 87.80 | 79.20 |
| TiO_2_ |  | N | 12.00 | 10.00 |
|  |  | Mean | 0.49 | 0.50 |
|  |  | Std | 0.16 | 0.21 |
|  |  | Min | 0.20 | 0.30 |
|  |  | Median | 0.50 | 0.45 |
|  |  | Max | 0.70 | 1.00 |
| LOI |  | N | 12.00 | 10.00 |
|  |  | Mean | 14.40 | 12.70 |
|  |  | Std | 7.63 | 3.51 |
|  |  | Min | 2.90 | 6.40 |
|  |  | Median | 13.45 | 12.95 |
|  |  | Max | 25.30 | 17.20 |
| Total elemental (XRF) analysis | | | | |
| As | (mg kg^-1^) | N | 12.00 | 10.00 |
|  |  | Mean | 8.49 | 5.96 |
|  |  | Std | 3.90 | 2.95 |
|  |  | Min | 2.50 | 2.50 |
|  |  | Median | 8.10 | 6.00 |
|  |  | Max | 14.43 | 11.43 |
| Ba |  | N | 12.00 | 10.00 |
|  |  | Mean | 747.58 | 667.03 |
|  |  | Std | 179.14 | 137.34 |
|  |  | Min | 465.41 | 512.07 |
|  |  | Median | 755.03 | 633.53 |
|  |  | Max | 1012.98 | 900.64 |
| Br |  | N | 12.00 | 10.00 |
|  |  | Mean | 29.89 | 11.00 |
|  |  | Std | 32.75 | 11.96 |
|  |  | Min | 3.04 | 2.30 |
|  |  | Median | 14.56 | 5.88 |
|  |  | Max | 105.89 | 41.90 |
| Co | (mg kg^-1^) | N | 12.00 | 10.00 |
|  |  | Mean | 10.87 | 13.77 |
|  |  | Std | 4.53 | 5.72 |
|  |  | Min | 3.51 | 8.71 |
|  |  | Median | 12.33 | 12.18 |
|  |  | Max | 16.40 | 24.37 |
| Cr |  | N | 12.00 | 10.00 |
|  |  | Mean | 93.98 | 91.90 |
|  |  | Std | 41.23 | 27.59 |
|  |  | Min | 37.06 | 52.91 |
|  |  | Median | 102.06 | 92.43 |
|  |  | Max | 176.68 | 129.20 |
| Cu |  | N | 12.00 | 10.00 |
|  |  | Mean | 30.03 | 28.30 |
|  |  | Std | 8.01 | 9.06 |
|  |  | Min | 15.75 | 13.80 |
|  |  | Median | 31.86 | 27.87 |
|  |  | Max | 40.48 | 41.27 |
| Nb |  | N | 12.00 | 10.00 |
|  |  | Mean | 3.81 | 3.77 |
|  |  | Std | 2.82 | 1.74 |
|  |  | Min | 0.50 | 0.50 |
|  |  | Median | 3.58 | 4.17 |
|  |  | Max | 7.94 | 6.00 |
| Ni |  | N | 12.00 | 10.00 |
|  |  | Mean | 31.49 | 38.64 |
|  |  | Std | 12.26 | 9.19 |
|  |  | Min | 12.27 | 24.62 |
|  |  | Median | 34.93 | 40.73 |
|  |  | Max | 44.98 | 50.33 |
| Pb |  | N | 12.00 | 10.00 |
|  |  | Mean | 10.64 | 11.95 |
|  |  | Std | 6.08 | 3.23 |
|  |  | Min | 1.00 | 7.15 |
|  |  | Median | 10.01 | 12.42 |
|  |  | Max | 18.90 | 17.79 |
| Rb |  | N | 12.00 | 10.00 |
|  |  | Mean | 61.70 | 61.60 |
|  |  | Std | 35.25 | 23.94 |
|  |  | Min | 23.40 | 40.02 |
|  |  | Median | 53.36 | 53.31 |
|  |  | Max | 114.58 | 104.06 |
| Sc |  | N | 12.00 | 10.00 |
|  |  | Mean | 9.49 | 7.60 |
|  |  | Std | 5.88 | 4.67 |
|  |  | Min | 1.00 | 1.00 |
|  |  | Median | 9.37 | 6.72 |
|  |  | Max | 24.09 | 16.81 |
| Sr | (mg kg^-1^) | N | 12.00 | 10.00 |
|  |  | Mean | 332.01 | 177.45 |
|  |  | Std | 403.74 | 113.97 |
|  |  | Min | 45.64 | 57.88 |
|  |  | Median | 152.94 | 157.04 |
|  |  | Max | 1187.53 | 407.60 |
| Th |  | N | 12.00 | 10.00 |
|  |  | Mean | 3.30 | 2.64 |
|  |  | Std | 3.07 | 1.46 |
|  |  | Min | 1.00 | 1.00 |
|  |  | Median | 1.56 | 3.20 |
|  |  | Max | 9.35 | 4.46 |
| V |  | N | 12.00 | 10.00 |
|  |  | Mean | 108.83 | 91.94 |
|  |  | Std | 50.99 | 34.99 |
|  |  | Min | 40.09 | 57.33 |
|  |  | Median | 104.29 | 81.73 |
|  |  | Max | 243.02 | 178.02 |
| Y |  | N | 12.00 | 10.00 |
|  |  | Mean | 15.37 | 16.70 |
|  |  | Std | 6.81 | 5.07 |
|  |  | Min | 7.53 | 10.52 |
|  |  | Median | 13.99 | 14.50 |
|  |  | Max | 25.04 | 24.22 |
| Zn |  | N | 12.00 | 10.00 |
|  |  | Mean | 48.99 | 50.55 |
|  |  | Std | 23.62 | 14.49 |
|  |  | Min | 12.52 | 31.90 |
|  |  | Median | 57.61 | 46.54 |
|  |  | Max | 79.72 | 74.28 |
| Zr |  | N | 12.00 | 10.00 |
|  |  | Mean | 208.37 | 203.79 |
|  |  | Std | 68.60 | 25.52 |
|  |  | Min | 120.32 | 169.49 |
|  |  | Median | 193.93 | 200.74 |
|  |  | Max | 348.28 | 245.54 |
