# Supplementary material for: Selected wetland soil properties correlate to Rift Valley fever livestock mortalities reported in 2009-10 in central South Africa
Source: PLoS One. 2020 May 18;15(5):e0232481. doi: 10.1371/journal.pone.0232481 (PMC7233588; doi:10.1371/journal.pone.0232481)
Supplement: Attachment — Submitted filename: Responce to Reviewers R2.pdf [file pone.0232481.s003.pdf]

20 March 2020

The Editor
PlosOne

RESPONSE TO REVIEW

Please accept herewith our revised submission (PONE-D-19-27989_R2). The manuscript has been significantly edited, taking the reviewers' comments into account. We sincerely apologise, especially to Reviewer 2. It was not our intention to offend, but rather ignorance on our part. To my knowledge, we have addressed all of the reviewers' comments. Please find our detail comments, in red font below.

Editor:

(1) The Intro section should be improved. From the beginning to Line 54, you provided sort of textbook knowledge only. Please write a more argumentative introduction, rather than an explanative one. Clearly discuss what knowledge gaps exist, why these gaps are important, what your hypothesis is, etc.

This was done.

(2) Line 118 -- It is very easy to follow Reviewer 1 and to provide a very simple description of "each master horizon." Please do so, rather than saying "everybody knows that."

This was done.

(3) The section titles "Physical and chemical analysis," "Mineralogical analysis," "Soil microbiology analysis," and "Active carbon analysis" could be combined to a single one, titled, for example "Soil analysis."

This was done.

(4) Line 186 -- Explain the van der Waerden analysis.

This was done.

(5) Lines 284-285 -- Why don't you present the results of pairwise correlation analysis between soil properties?

This was done. (Table 4)

Reviewer #1:

There is a discrepancy between the described results and the discussion with different variables being described as being included in the final model. (Line numbers refer to the marked-up version). On line 255 of the marked-up version the results clearly state the following "four variables (exchangeable K+, exchangeable Mg2+, medium sand, and Br) were identified by the stepwise variable selection of the discriminant analysis to discriminate between sites with and without reported RVF mortality". There is a clear list of FOUR variables. However, in the discussion line 275 the authors refer to three variables "soluble Ca2+ and exchangeable K+, and medium sand". The authors are discussing different variables from their results – soluble Ca2+ has been added and Br and exchangeable Mg2+ dropped? Then again on line 290 they refer to four variables being in the model "Although only soluble Ca2+, exchangeable K+, Br and medium sand were used in the final model". Again a different list of variables. Please note that soluble Ca2+ is not described in the results in page 15 as being in the final model. In the conclusion line 333-335 the authors again refer to three variables "Three soil characteristics (soluble Ca2+, exchangeable K+, and medium sand) were subsequently

selected for inclusion into a discriminant function that could potentially be used to predict an RVF outbreak site" In the abstract line 11-12 the authors write "Three soil characteristics (soluble Ca2+, exchangeable K+, and medium sand) were consequently included in a discriminant function that could potentially be used to predict a Rift Valley fever outbreak site". This inconsistency regarding the number of variables and the list of variables included in the final model NEEDS to be rectified before publication.

This was corrected.

Minor amendments

The authors might colour the sampling sites more clearly in Figure 1

I have used un-filled triangles to indicate the study sites, because they overlap at the scale of presentation. I therefore did not change the current layout, although I can easily do so if required.

Line 172 This sentence starts with a digit "2.5g" and should be rewritten

This was corrected.

Line 178 measureing is misspelled

This was corrected.

Line 297 and Line 298 there appears to be issues with the references?

This was corrected. I have also checked the other references, since more such issues were found. It seems that the cross references went awry during the editing and by using track changes.

Reviewer #2:

1) The submission has been made to PLoS One---not the Journal of Soil Sciences. Therefore, the authors' assertion that certain terminology "is common knowledge for anyone working with soil" is inappropriate. Comments like these generally don't help your argument; and to be honest, the Reviewer finds it to be quite rude. The authors should appreciate that comments like these may automatically get your manuscript rejected.

I hereby offer my sincere, unreserved apology and assure you that no affront was intended.

2) Just because the second Reviewer does't repeat a concern that the first Reviewer raises, it doesn't mean that the first Reviewer's concerns are not justified. As the second reviewer, I actually agree with Reviewer #1 on most points (including points 9 and 17); and just for your information, Reviewer # 2 also had to look up the definition of "Master Horizon". This is simply another example of poor conduct on the part of the authors.

We will gladly include any elucidation required, but there is also an assumed common discourse, which in this case obviously did not hold true. Once again, my sincere apologies.

Kind regards

CW van Huyssteen
