# Supplementary material for: Selected wetland soil properties correlate to Rift Valley fever livestock mortalities reported in 2009-10 in central South Africa
Source: PLoS One. 2020 May 18;15(5):e0232481. doi: 10.1371/journal.pone.0232481 (PMC7233588; doi:10.1371/journal.pone.0232481)
Supplement: Attachment — Submitted filename: Response to Reviewers_R3.pdf [file pone.0232481.s004.pdf]

UNIVERSITY OF THE
FREE STATE
UNIVERSITEIT VAN DIE
VRYSTAAT
YUNIVESITHI YA
FREISTATA

UFS•UV
NATURAL AND AGRICULTURAL SCIENCES
NATUUR- EN LANDBOUWETENSKAPPE

14 April 2020

The Editor
PlosOne

RESPONSE TO REVISION R2

Please accept herewith our revised submission (PONE-D-19-27989_R3). The following revisions were made:

Reviewer #1:

(I give the line numbers of R3 in brackets.)

1. Line 54 (56): “with with” changed to “with”
2. Line 86 (88): “Bace” changed to “Base”
3. Line 159 (161/220): The nomenclature as used is in fact correct. We report on four layers’ data: Layer 1 represented the 0-50 mm layers; Layer 2 represented the A horizons; Layer 3 represented the B/B1/G/C1 horizons; and Layer 4 represented the B2/C/C2 horizons.

Reviewer #2:

No corrections suggested.

Kind regards

CW van Huyssteen

205 Nelson Mandela Drive, Park West, Bloemfontein 9301, South Africa
Soil Science (54), PO Box 339, Bloemfontein 9300, South Africa
